# Diagnostic performance of intraoperative assessment in grade 2 endometrioid endometrial carcinoma

**DOI:** 10.1186/s12957-020-02056-7

**Published:** 2020-10-30

**Authors:** Antonio Bandala-Jacques, David Cantú-de-León, Delia Pérez-Montiel, Rosa A. Salcedo-Hernández, Diddier Prada, Aarón González-Enciso, Arely Gonzalez-Valdés, Salim Abraham Barquet-Muñoz

**Affiliations:** 1 Biomedical Cancer Research Unit, Instituto Nacional de Cancerología, Mexico City, Mexico; 2 Biomedical Investigations Institute, Universidad Nacional Autonóma de México, Mexico City, Mexico; 3 Instituto Nacional de Cancerología, Mexico City, Mexico; 4 Department of Pathology, Instituto Nacional de Cancerología, Mexico City, Mexico; 5 Department of Gynecology, Instituto Nacional de Cancerología, Mexico City, Mexico; 6 Department of Biomedical Informatics, Faculty of Medicine, Universidad Nacional Autónoma de México, Mexico City, Mexico; 7 Department of Environmental Health Sciences, Mailman School of Public Health, Columbia University, New York City, USA; 8 Department of Surgery, Instituto Nacional de Cancerología, Mexico City, Mexico

**Keywords:** Endometrial cancer, Endometrioid adenocarcinoma, Surgical diagnostic technique, Frozen sections, Lymphadenectomy, Myometrial invasion

## Abstract

**Abstract:**

**Background:**

Endometrial carcinoma is the most common gynecologic malignancy in developed countries. Grade 2 carcinoma is associated with pelvic lymph-node metastasis, depending on selected risk factors. Intraoperative assessment (IOA) can identify patients at risk for lymph node metastasis who should undergo staging surgery. Our objective was to establish the diagnostic precision of IOA in determining the need for surgical staging in grade 2 endometrioid endometrial carcinoma.

**Methods:**

Two hundred twenty-two patients underwent IOA. Results were compared to the final pathology report. The accuracy of the IOA parameters was calculated. Variables were evaluated in patients with positive versus negative IOA. Overall and disease-free survivals were calculated according to IOA, lymphadenectomy, and nodal metastasis.

**Results:**

IOA was positive in 80 patients. It showed an accuracy of 76.13% when compared with the postoperative assessment. The best individual parameter was myometrial invasion. Nodal metastasis was observed in 16 patients in the positive IOA group and 7 patients in the negative group. Patients with lymph node metastasis had a 5-year overall survival rate of 80.9%, whereas patients without metastasis had a 5-year overall survival rate of 97.9%.

**Conclusions:**

IOA is an adequate tool to identify high-risk patients in grade 2 endometrial carcinoma. Myometrial invasion is the individual parameter that yields the highest diagnostic precision.

## Background

Endometrial carcinoma is the most common gynecologic malignancy in high- and middle-income countries [1]. It is the fourth most common cancer in women and has had a rising incidence trend in the last decade [2]. The most common histology is endometrioid, and its treatment consists of hysterectomy with bilateral salpingo-oophorectomy [3]. Patients may undergo pelvic and paraaortic lymphadenectomy depending on their risk for lymph node metastasis. Lymph node metastasis is one of the most important prognostic factors for endometrial cancer, as it renders a tumor with a high risk for recurrence that calls for adjuvant therapy [4].

Lymph node metastasis cannot be determined without node dissection, especially if metastasis is microscopic, but it strongly associates with a high tumor grade and deep myometrial invasion, which can be determined intraoperatively, through intraoperative gross examination, frozen section biopsy, and/or touch imprint cytology [5]. If the intraoperative assessment (IOA) shows high-risk factors, complete surgical staging is recommended [6]. That is, as pelvic and paraaortic lymphadenectomy procedures increase morbidity and the surgical time, they are not performed routinely [7]. Furthermore, the final pathology report can differ from intraoperative findings, and IOAs could lead to unnecessary surgeries in women with low-risk disease [8].

According to the 2009 FIGO staging system, grade 2 endometrial carcinomas are defined as tumors with, from 6 to 50% of solid non-glandular, non-squamous growth architectural elements [9]. Grade 2 tumors are considered at low to medium risk for node metastasis, especially when combined with other factors, such as the depth of myometrial invasion and lymphovascular invasion [10, 11]. For example, grade 2 endometrioid carcinomas with less than 50% myometrial invasion have a 4.8% probability of lymph node metastasis [12]. However, in the case of grade 2 endometrioid tumors that infiltrates more than 50% of the myometrium, the probability of pelvic lymph node metastasis increases to 15% [13].

Therefore, an accurate judgment of the elements in the IOA is important for deciding treatment strategies. The evaluation of multiple parameters together, such as grade and myometrial, cervical, or ovarian involvement, may prove accurate in discriminating high- from low-risk patients for lymph node metastasis [14]. Patients with a high risk for lymph node metastasis should undergo lymphadenectomy, while those with a low risk can be spared the risks and complications of a staging surgery.

The purpose of this study was to establish the precision of intraoperative assessment in determining the need for surgical staging in grade 2 endometrioid endometrial carcinoma of the uterus.

## Materials and methods

Data used in this study was obtained from electronic hospital records of patients treated at our institution between January 2016 and December 2018. The protocol was approved by our Institutional Review Board, with approval reference Rev/020/20. The inclusion criteria were women older than 18 years with grade 2 endometrioid endometrial carcinoma as confirmed by our institution’s Pathology Department that underwent surgical intraoperative assessment. The exclusion criteria were incomplete data, non-endometrioid histology, or final pathology report of a histological grade other than two.

Patients were analyzed according to intraoperative assessment positivity. Intraoperative assessment consisted of gross examination and frozen section biopsy. A positive IOA was defined as myometrial invasion greater than 50% in depth or with cervical, serous, or ovarian involvement. Disease-free survival was defined as the time frame between surgical treatment and disease recurrence or the date last seen. Overall survival was defined as the time frame between diagnosis and death or the date last seen.

Patients underwent lymphadenectomy in accordance with both the IOA results and clinical criteria as determined by gynecologic oncologists, such as enlarged lymph nodes with abnormal consistency or conglomerated retroperitoneal lymph node masses. Pelvic lymphadenectomy consisted of the bilateral dissection of all lymph nodes between the circumflex vein as the inferior margin, 2 cm above the bifurcation of the external iliac artery as the superior margin, the genitocrural nerve as the lateral margin, the superior vesical artery as the medial margin, and the obturator nerve as the dissection floor. Paraaortic lymphadenectomy had ureters as the lateral margins, the infrarenal vein as the superior margin, and 2 cm above the external iliac artery bifurcation as the inferior margin.

### Statistical analysis

For the descriptive analysis, the Shapiro-Wilk test was used to identify the normality of the continuous variables. The mean and standard deviation (SD) were used for continuous normal variables, and the median and interquartile range (IQR) were used for continuous non-normal variables. Absolute and relative frequencies were used for categorical variables. For the comparative analysis, Student’s *t* test, Wilcoxon’s rank-sum test, the chi-squared test, or Fisher’s exact test were used depending on the analyzed variable. Survival curves were generated with Kaplan-Meier estimator and compared with the log-rank test. Logistic regression was used to obtain the odds ratios (ORs) and establish factors associated with frozen section biopsies. Diagnostic tests with the area under the curve (AUC), sensitivity, specificity, and predictive values were performed to estimate the diagnostic value of intraoperative frozen section biopsy, taking the definitive pathology report as the reference standard. Statistical significance was defined as a *p* value < 0.05. Statistical analysis was performed with the STATA software, version 13.0 (College Station, TX, licensed to the author).

## Results

A total of 222 patients met the inclusion criteria and were analyzed. The mean patient age was 54.5 years (SD 11.7). The median body mass index was 31 (IQR 27.1-35.9) kg/m^2^. Postoperative stages were I in 162 (73%) patients, II in 25 (11.2%), III in 30 (13.5%), and IV in 5 (2.2%). Eighty-seven (39.2%) patients underwent lymphadenectomy.

All patients had an IOA. Frozen section biopsy was positive in 80 (36%) patients. The IOA was positive for ovarian involvement in 2 (0.9%) patients, uterine serosa involvement in 12 (5.4%), and cervical involvement in 29 (13%). Myometrial invasion was superficial in 34 (15.3%) patients, less than 50% deep in 137 (61.7%), and greater than 50% deep in 51 (23%). The presurgical tumor grade was grade 1 in 7 (11.1%) patients, grade 2 (73%) in 46 patients, and grade 3 in 9 (14.3%) patients. In the final pathology report, the median tumor size was 40 mm (IQR 30-35), there was ovarian involvement in 10 (4.5%) patients, uterine serosa involvement in 3 (1.4%), and cervical involvement in 55 (24.8%). Myometrial invasion was superficial in 34 (15.3%) patients, less than 50% gross depth invasion in 133 (59.5%), and greater than 50% gross depth invasion in 55 (24.8%). There was lymph node metastasis in 23 (10.4%) patients (Table 1).

**Table 1:** Tumor characteristics as reported in the intraoperative assessment and final pathology report, *n* = 222

Intraoperative assessment report	
Positive intraoperative assessment	80 (36%)
Myometrial invasion	
Superficial	34 (15.3%)
≤ 50%	137 (61.7%)
> 50%	51 (23%)
Cervical involvement	29 (13%)
Uterine serosa involvement	12 (5.4%)
Ovarian involvement	2 (0.9%)
Grade, *n* = 63 (28.37%)	
1	7 (11.1%)
2	47 (74.6%)
3	9 (14.3%)
Final pathology report	
Tumor size, mm	40 (30-35)^a^
Myometrial invasion	
Superficial	34 (15.3%)
≤ 50%	133 (59.9%)
> 50%	55 (24.8%)
Uterine serosa involvement	3 (1.4%)
Ovarian involvement	10 (4.5%)
Cervical involvement	55 (24.8%)
Lymphovascular permeation	41 (18.5%)
Lymph node metastasis (*n* = 89)	
Yes	23 (10.4%)
No	66 (29.7%)

^a^Median (interquartile range)

The IOA showed an accuracy of 76.1% when compared with the postoperative assessment. It had an AUC of 0.74 (95% confidence interval [CI] 0.68-0.8), a sensitivity of 65.5%, a specificity of 83%, a positive predictive value of 71.3%, and a negative predictive value of 78.9%. Myometrial invasion had an AUC of 0.76 (95% CI 0.69-0.83) and an accuracy of 82.4% when compared with myometrial invasion in the final pathology report. Cervical involvement had an AUC of 0.61 (95% CI 0.55-0.68) and an accuracy of 77.5%. Uterine serosa involvement had an AUC of 0.47 (95% CI 0.46-0.49) and an accuracy of 93.2%. Ovarian involvement had an AUC of 0.55 (95% CI 0.45-0.65) and an accuracy of 95.5%. The rate of lymph node metastasis according to each parameter’s positivity was 25.5% for myometrium, 13.8% for cervix, 33.3% for uterine serosa, and 50% for ovary (Table 2).

**Table 2:** Diagnostic accuracy of intraoperative assessment parameters

	*N* (%)	LN metastasis rate (%)	AUC (95% CI)	Sen	Spe	PPV	NPV	LR+	LR−	Accuracy
Overall IOA +	80 (36)	16 (20)	0.74 (0.68-0.80)	65.5	83	71.3	78.9	3.9	0.42	76.1
Myometrium IOA+	55 (24.8)	14 (25.5)	0.76 (0.69-0.83)	64.3	88.6	65.4	88	5.6	0.4	82.4
Cervix IOA +	29 (13)	4 (13.8)	0.61 (0.55-0.68)	30.9	92.8	58.6	80.3	4.3	0.74	77.5
Serous IOA +	12 (5.4)	4 (33.3)	0.47 (0.46-0.49)	0	94.5	0	98.6	0	1.5	93.2
Ovarian IOA +	2 (0.9)	1 (50)	0.55 (0.45-0.65)	10	99.5	50	95.9	21.2	0.9	96

*AUC* area under the curve, *Sen* sensitivity, *Spe* specificity, *PPV* positive predictive value, *NPV* negative predictive value, *LR* likelihood ratio, *IOA* intraoperative assessment

A total of 80 (36%) patients had positive IOA, and 142 (64%) were negative. Lymphadenectomy was performed on 69 (86.3%) patients in the positive IOA group and 18 (12.7%) in the negative IOA group (*p* < 0.001). There was lymph node metastasis in 16 (20%) patients in the positive IOA group and 7 (4.9%) patients in the negative IOA group (*p* < 0.001). Regarding intraoperative complications, patients in the positive IOA group had more intraoperative bleeding (375 ml, IQR 80-300) than those in the negative IOA group (150 ml, IQR 200-550) (*p* < 0.001). Likewise, there were 8 (10%) blood transfusions in the positive frozen section biopsy group and none (0%) in the negative frozen section biopsy group (*p* < 0.001). There were no differences found regarding age, menarche, menopause, weight, reintervention, stage IV disease, or ICU admission (Table 3).

**Table 3:** Comparative analysis according to the intraoperative assessment results, *n* = 222

	IOA negative	IOA positive	*P*
	142 (64%)	80 (36%)	
Age^a^	53.7 ± 11.4	56 ± 12.2	0.166
Menopause	108 (76%)	62 (77.5%)	0.807
Weight, kg^b^	74.5 (65.5-85.5)	68.5 (60-80.5)	0.079
BMI^a^	32.6 ± 6.7	31.1 ± 7.1	0.131
Surgical stage			
I	120 (84.5%)	42 (52.5%)	< 0.001
II	12 (8.5%)	13 (16.3%)	
III	8 (5.6%)	22 (27.5%)	
IV	2 (1.4%)	3 (3.8%)	
Lymph node metastasis	7 (4.9%)	16 (20%)	< 0.001
Lymphadenectomy	18 (12.7%)	69 (86.3%)	< 0.001
Bleeding, ml^b^	150 (80-300)	375 (200-550)	< 0.001
Transfusion	0 (0%)	8 (10%)	< 0.001
Reintervention	2 (1.4%)	3 (3.8%)	0.277
ICU	2 (1.4%)	5 (6.3%)	0.07
Adjuvant therapy	44 (31%)	62 (77.5%)	< 0.001
Radiotherapy	41 (28.9%)	58 (72.5%)	0.94
Chemotherapy	8 (5.6%)	22 (27.5%)	0.049
Recurrence of disease	7 (4.9%)	8 (10%)	0.156

*FSB* frozen section biopsy, *BMI* body mass index, *ICU* intensive care unit, *IOA* intraoperative assessment

^a^Mean ± standard deviation

^b^Median (interquartile range)

**Fig. 1:**
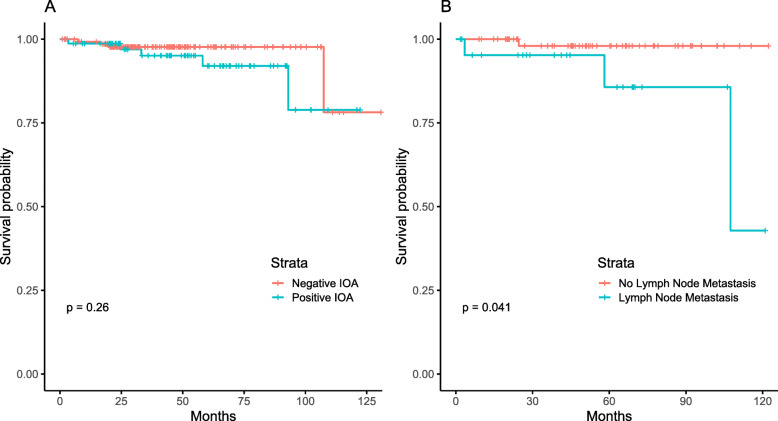
Overall survival according to intraoperative assessment (*p* = 0.257) and lymph node metastasis (*p* = 0.04)

The median follow-up duration was 43.8 (IQR 24.47-65.8) months. The 5-year overall survival rate for all patients was 95.3% (95% CI 89.5-97.9). Patients with a positive IOA had a 5-year overall survival rate of 92% (95% CI 79.05-97.1), whereas patients with a negative IOA had a 5-year overall survival rate of 97.7% (95% CI 93.08-99.26) (*p* = 0.257). Patients who underwent lymphadenectomy had a 5-year overall survival rate of 94.7% (95% CI 83.4-98.4), whereas those who did not undergo lymphadenectomy had a 5-year overall survival rate of 96.2% (95% CI 89.8-98.6) (*p* = 0.99). Patients with lymph node metastasis had a 5-year overall survival rate of 80.9% (95% CI 65.3-96.5), whereas patients without lymph node metastasis had a 5-year overall survival rate of 97.9% (95% CI 86.4-99.7) (*p* = 0.04) (Fig. 1).

**Fig. 2:**
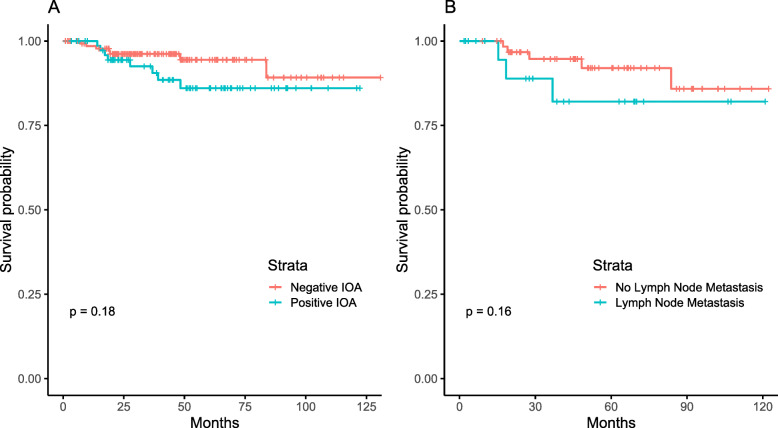
Disease-fee survival according to intraoperative assessment (*p* = 0.177) and lymph node metastasis (*p* = 0.159)

The 5-year disease-free survival rate was 91.3% for all patients. Patients with a positive IOA had a 5-year disease-free survival rate of 86% (95% CI 73.5-92.91), whereas patients with a negative IOA had a 5-year disease-free survival rate of 94.4% (95% CI 87.29-97.62) (*p* = 0.177). Patients who underwent lymphadenectomy had a 5-year disease-free survival rate of 91.1% (95% CI 80.94-96), whereas patients who did not undergo lymphadenectomy had a 5-year disease-free survival rate of 91.3% (95% CI 82.4-95.9) (*p* = 0.789). Patients with lymph node disease had a 5-year disease-free survival rate of 91.3% (95% CI 82.4-95.8), whereas patients without lymph node disease had a 5-year disease-free survival rate of 93.9% (95% CI 81.6-98) (Fig. 2).

## Discussion

The main findings of our study were that in patients with grade 2 endometrioid endometrial carcinomas, IOA is an adequate tool to determine the need for lymph node evaluation. Out of the IOA parameters, myometrial invasion had the greatest diagnostic precision. Furthermore, undergoing the procedure does not impact overall survival or disease-free survival.

The usefulness of an IOA is debatable, as its misinterpretation can lead to unnecessary surgeries [15]. In our study, the IOA had a 76% concordance rate with the final pathology report, with the best overall predictor being myometrial invasion. Studies have found myometrial invasion to be a good predictor of node metastasis, with a sensitivity as high as 86% [7]. Our overall accuracy for myometrial invasion was 82%. While cervical involvement had a precision of 77%, it had a low sensitivity (30%). This might be because the IOA is not able to detect microscopic cervical foci, which can be detected only in the final postoperative assessment [16]. Although there is great variation in the reported concordance of the IOA with the final pathology report between institutions, an analysis of both by a trained gynecologic pathologist improves the accuracy of the IOA [17].

Incomplete surgical staging can have consequences such as extended-field radiotherapy or further staging procedures if the final pathology report demonstrates high risk [18]. Grade determination by curettage alone is unreliable, as it can differ from the final report in over 50% of cases, and other techniques such as magnetic resonance imaging are not always available in all centers. A gross evaluation of the uterus improves this accuracy [19]. A study found a concordance rate of 86% between frozen sections and paraffin sections for grade 2 tumors, which was lower than those for grade 1 or 3 tumors. The study also found an underestimation of 8.2% [20]. Our institution does not always include tumor grade in the presurgical report, but of those available, there is 75% concordance and 11% underestimation.

After IOA has identified patients at risk for lymph node metastasis, they can undergo lymphadenectomy. Lymphadenectomy plays only a diagnostic role, does not impact the prognosis, and does not increase the risk of intraoperative complications [21]. Our study confirmed that pelvic and paraaortic lymphadenectomy in grade 2 endometrioid endometrial carcinoma had no impact on overall survival or disease-free survival and was not associated with a higher risk of intraoperative complications. However, lymphadenectomy did help identify patients with lymph node disease, which does impact survival [22]. Lymph node metastasis significantly affects disease prognosis and can reduce 5-year overall survival from as high as 91% to as low as 44% [23]. In our study, having positive lymph nodes reduced the 5-year overall survival rate from 97.9 to 85.7%. A different study found a reduction in the 5-year overall survival rate from 96.5 to 77.6% in the presence of pelvic node metastasis and worse outcomes with paraaortic node metastasis [24]. As this is an important prognostic factor, it is critical to identify patients with lymph node metastasis. Even with novel techniques, the use of IOA with gross examination and frozen section is widespread and can help determine high-risk factors that warrant surgical staging with lymphadenectomy. On the other hand, sometimes patients with negative frozen section biopsies undergo lymphadenectomy if there is high clinical suspicion of node metastasis. In our study, 18 out of the 142 patients with a negative frozen section received lymphadenectomy, out of which 7 (38.9%) were positive for node metastasis. If clinical suspicion remains high after a negative IOA, patients should undergo lymphadenectomy regardless.

Sentinel lymph node mapping has become an increasingly popular alternative for lymphadenectomy due to its shorter operative time and reduced number of lymphatic complications [25]. Although it is now also included as a standard technique for nodal evaluation, large cohorts have found that its survival rate is similar to that of systematic lymphadenectomy [26]. As sentinel lymph node biopsy is not yet widely available in low-income countries, it is safe to continue comprehensive lymphadenectomy with frozen sections if available.

The limitations of our study include its retrospective nature. Furthermore, our institution does not report tumor grade on most IOAs, so we were unable to evaluate tumor grade mismatch in most patients. The main strength of our study is that it provides insights of the performance of a widespread diagnostic technique when applied exclusively to grade 2 tumors, and that it was performed in an oncologic institution where specialized gynecologic pathologists review all specimens.

## Conclusions

In conclusion, our study supports the use of IOA with gross examination and frozen section biopsy for determining the need for lymph node evaluation by lymphadenectomy in patients with grade 2 endometrioid endometrial carcinomas. Within the IOA, myometrial invasion yields the highest overall diagnostic accuracy. Patients with negative frozen sections should undergo lymphadenectomy regardless if clinical suspicion remains high. Lymphadenectomy does not affect disease-free survival or overall survival, but it does help to identify patients with node metastasis.

## Data Availability

The datasets generated and/or analyzed during the current study are not publicly available due to hospital policy but are available from the corresponding author on reasonable request.

## Abbreviations

IOA: Intraoperative assessment
AUC: Area-under-curve
